# Avian Influenza A Virus Polymerase Recruits Cellular RNA Helicase eIF4A3 to Promote Viral mRNA Splicing and Spliced mRNA Nuclear Export

**DOI:** 10.3389/fmicb.2019.01625

**Published:** 2019-07-16

**Authors:** Xingxing Ren, Yuandi Yu, Huanan Li, Jinyu Huang, Aobaixue Zhou, Shukai Liu, Pingsheng Hu, Bo Li, Wenbao Qi, Ming Liao

**Affiliations:** ^1^National Avian Influenza Para-Reference Laboratory (Guangzhou), College of Veterinary Medicine, South China Agricultural University, Guangzhou, China; ^2^National and Regional Joint Engineering Laboratory for Medicament of Zoonoses Prevention and Control, College of Veterinary Medicine, South China Agricultural University, Guangzhou, China; ^3^Key Laboratory of Zoonosis, Ministry of Agriculture, College of Veterinary Medicine, South China Agricultural University, Guangzhou, China; ^4^Key Laboratory of Zoonosis Prevention and Control of Guangdong Province, Guangzhou, China

**Keywords:** avian influenza A virus, PB2, eIF4A3 RNA helicase, polymerase, splicing, mRNA export

## Abstract

The influenza A virus replicates in a broad range of avian and mammalian species by hijacking cellular factors and processes. Avian influenza A viruses (AIVs) generally propagated poorly in mammalian cells, but some mutants of virus-encoded RNA polymerase components, especially PB2 subunit, can overcome host restriction. Host factors associated with PB2 may be essential for efficient AIV replication in mammalian cells. Here, we infected human cells with the PB2 Flag-tagged replication-competent recombinant AIV and identified cellular proteins that coprecipitate with PB2 protein by mass spectrometry. We confirmed one of the coprecipitating host factors, DEAD-box protein eIF4A3, that interacts with viral PB2, PB1, and NP proteins. Depletion of endogenous eIF4A3 significantly reduced virus replication. Later studies showed that eIF4A3 is essential for viral RNA polymerase activity and viral RNAs synthesis. Upon systematic dissection of the influenza virus progeny mRNA generation, from pre-mRNA processing to nuclear export, we found that the depletion of eIF4A3 resulted in significant defects in the ratio of M2 to M1 and NS2 to NS1, and the proportion of viral spliced mRNA in the nucleus increased, indicating that eIF4A3 plays a significant function in viral nascent intron mRNA splicing and spliced mRNA (M2 and NS2) nuclear export. Additionally, we confirmed that in specific deletion of eIF4A3, the synthesis of reduced NS2 can significantly impair neo-synthetized viral ribonucleoprotein (vRNP) nuclear export. Taken together, our findings revealed that eIF4A3 is a key mediator of AIV polymerase activity, mRNA splicing, and spliced mRNA nuclear export.

## Introduction

Influenza A virus is a single-stranded, segmented, negative-sense RNA virus in the *Orthomyxoviridae* family that causes a highly contagious respiratory disease in humans and animals (Nelson and Holmes, 2007). To date, pathogenic strains of influenza A viruses have led to several serious pandemics and caused high mortality rates, for example, the pandemics of 1918, 1957, 1968 and 2009 (Medina, 2018). Avian influenza A viruses (AIVs) do not usually infect humans, but many, typically severe cases of human infection with H5, H6, H7, H9, and H10 subtype AIVs have been reported since 1997 (Li et al., 2004; Paules and Subbarao, 2017; Shi et al., 2018). High mutation rates of influenza virus can escape host immune response. For this reason, vaccines and drugs that act directly on viruses are potentially ineffective (Osterholm et al., 2012). Hence, there is a pressing need for a better understanding of the influenza virus replication mechanism to find novel potential antiviral targets.

The genome of the AIV is composed of eight negative-sense RNA segments coding for at least 11 proteins. Each viral RNA (vRNA) segment is wrapped with multiple copies of oligomeric nucleoprotein (NP) and together with the heterotrimeric virus-encoded, RNA-dependent RNA polymerase (RdRP, were composed of PB2, PB1, and PA proteins) formed viral ribonucleoprotein complexes (vRNPs) (Pflug et al., 2014). Once in the infected cells, the vRNPs are released and transported to the nucleus, where they undergo transcription and replication. The PB2 and PA provide the 5′ capped RNA primers for vRNA transcription by binding to and cleaving capped host pre-mRNA, respectively. The PB1 is the core of polymerase, catalyzing the sequential chain elongation (Te Velthuis and Fodor, 2016). Moreover, the function that viral polymerase performs requires the participation of a large amount of host cell resources. For instance, the cellular RNA polymerase II (Pol II) associated with viral neonatal mRNA capping and splicing (Fodor, 2013), the host kinase PKC phospho-regulation of NP oligomerization and RNP assembly (Mondal et al., 2017). AIVs generally propagate poorly in mammalian cells. Many previous studies, including our previous findings, indicated that some amino acid substitution on the PB2 subunit of the polymerase, such as E627K, D701N, and A588V, can greatly improve the AIV polymerase activity in mammalian cells, thus increasing the adaptability of viruses in mammals (Hatta et al., 2001; Le et al., 2009; Xiao et al., 2016). Some host factors that interact with PB2, have been proven to be involved in this adaptive mechanism, for instance, importin alpha, ANP32A, and RIG-I (Hudjetz and Gabriel, 2012; Weber et al., 2015; Long et al., 2016). However, despite that, we have very limited knowledge of which cellular factors are involved in virus replication (Shaw, 2011). Therefore, understanding the role of cellular factors in the influenza polymerase function process is critical and may provide better prophylactic and therapeutic treatments against influenza infection.

The eukaryotic initiation factor 4A isoform 3 (eIF4A3) is an archetypical member of the DEAD-box RNA helicase family, which is mainly located in the nucleus, and in company with Y14, Magoh, and MLN15 forms the core of the splicing-dependent exon-exon junction complex (EJC), mainly associated with nuclear mRNA export, subcellular mRNA localization, translation efficiency, and nonsense-mediated mRNA decay (NMD) (Chan et al., 2004). During post-transcriptional stages, the spliceosome-associated protein CWC22 recruits eIF4A3 to the spliceosome. Then, Y14-Magoh heterodimers were recruited to the activated spliceosome to bind eIF4A3 stably. Upon exon ligation and release, eIF4A3-Y14-Magoh protein complex bound to messenger RNA 24 nt upstream of exon-exon junctions and exercised splicing functions (Singh et al., 2012). After completion of splicing, EJC interacts with the export adapter that promotes spliced mRNP export to the cytoplasm through the nuclear pore complex (Le Hir et al., 2016).

Unlike most intronless viruses, two of the influenza A virus mRNA segments, M and NS, generate the spliced product. The NS segment produces two overlapping sequences from the same post-transcription mRNA. NS1 is encoded by conventional transcript in the NS segment with an open reading frame (ORF) 864 nucleotides, whereas the NS segment was interrupted by splicing machinery from position 57 to 526 producing NS2/NEP mRNA. As with the NS segment, the M segment yield unspliced M1, spliced M2, and spliced mRNA_3_ mRNA. In addition, the spliced viral mRNA (M2, NS2) has a distinct mRNA export approach compared with the intronless mRNA in influenza A virus (Dubois et al., 2014). Virus exploits the host RNA helicase eIF4A3 to promote spliced viral mRNA formation and nuclear export has been confirmed in the life cycle of human cytomegalovirus (HCMV) and herpes simplex virus (HSV) (Sadek and Read, 2016; Ziehr et al., 2016), however, the role of eIF4A3 in the influenza A virus spliced viral mRNA process has never been reported.

Several high-throughput screening methods have identified numbers of host factors that may be involved in influenza virus replication and cross-host propagation, including eIF4A3 (Karlas et al., 2010; Tripathi et al., 2015). However, the specific function of eIF4A3 in influenza virus replication has not been studied. In the present study, we have taken a proteomic approach which identified and confirmed that eIF4A3 interacted with influenza A virus PB2, PB1, and NP proteins in mammalian cells. We also validated that the depletion of endogenous eIF4A3 by siRNA knockdown decreased viral RNA polymerase activity and virus replication. Through functional analyses, we verified that eIF4A3 was essential for the splicing of M and NS mRNA and participating in the nuclear export of spliced mRNA (M2 and NS2). Thus, eIF4A3 was revealed as one of the key mediators that participate in the AIV life cycle.

## Materials and Methods

### Cells and Viruses

Human epithelial A549, HEK-293T, MDCK, and HeLa cells were maintained in Dulbecco’s minimal essential medium (DMEM, Gibco) supplemented with 10% fetal bovine serum (FBS, Biological Industries, Israel), 100 U/mL penicillin, and 100 U /mL streptomycin, at 37°C in 5% CO_2_. Influenza A/Chicken/Guangdong/V/2008 (H9N2) virus was produced and titrated as previously described (Li et al., 2012).

### Antibodies

Hybridoma cells secreting mouse monoclonal anti-influenza A virus (IAV) NP (clone H16-L10-4R5, ATCC HB-65) and anti-IAV M (M2-1C6-4R3, ATCC HB-64) were obtained from ATCC and antibodies in the supernatant purified using a protein G column (Yewdell et al., 1981). Other antibodies used include: rabbit monoclonal anti-eIF4A3 (ab180573, Abcam), mouse monoclonal anti-IAV NP (M100014, Zoonogen, China), rabbit polyclonal anti-IAV M1(GTX125928, GenTex), rabbit polyclonal anti-IAV M2 (GTX125951, GenTex), rabbit polyclonal anti-IAV NS1(GTX125990, GenTex), rabbit polyclonal anti-IAV NS2 (GTX125953, GenTex), anti-FLAG M2 Affinity Gel (A2220, Sigma), anti-Strep-Tactin Sepharose (2-1201-010, IBA), mouse monoclonal anti-FLAG M2 (F3165, Sigma), mouse monoclonal Anti-Strep Tag (SAB2702216, Sigma), mouse monoclonal anti-Myc tag (AF0033, Beyotime), mouse monoclonal anti-GAPDH (HC301, Transgen), mouse monoclonal anti-Histone H2B (AH426, Beyotime), IRDye 800CW goat anti-rabbit IgG (926-32211, LC-COR), IRDye 800CW goat anti-mouse IgG (926-32210, LC-COR), FITC conjugated goat anti-mouse IgG (HS211, Transgen), Alexa Fluor 594 conjugated goat anti-rabbit IgG (H+L) (A-11012, Invitrogen).

### Plasmids

The eukaryotic expression vector pPRE was kindly provided by Dr. Feng Li (South Dakota State University, United States); this plasmid was used under the control of both the cytomegalovirus (CMV) immediate-early promoter and the bovine growth hormone polyadenylation signal (Wang et al., 2010). The pPRE-eIF4A3-Strep and pPRE-eIF4A3-Myc were constructed by digesting pPRE-Strep or pPRE-Myc plasmids with *Xba*I and *Xho*I restriction endonucleases. Full-length human eIF4A3 cDNA was amplified by PCR cDNA prepared from total RNA of HEK-293T cells as the template (Forward primer: 5′-CTAGTCTAGA ATGGCGACCA CGGCCACGA-3′, Reverse primer: 5′-CCGCTCGAGG ATAAGATCAG CAACGTTCA-3′). Strep-tagged eIF4A3 and Myc-tagged eIF4A3 were constructed in expression vector pPRE with a Strep or Myc tag at the C-terminus. The sequences coding for PB2, PB1, PA, and NP proteins derived from the influenza A/Chicken/Guangdong/V/2008 (H9N2) virus were amplified by RT-PCR. Primer sequences used are listed as follows (5′-3′), PB2 (Forward: ATCTCGAGAT GGAGAGAAT AAAAGAAT, Reverse: GTGCGGCCGC TTAATTGATG GCCATCCGA), PB1 (Forward: ATCTCGAGAT GGATGTCAAT CCGACTC, Reverse: GTGCGGCCGC TTATTTTTGC CGTCTGAGC), PA (Forward: ATCTCGAGAT GGAAGACTTT GTGCGAC, Reverse: GTGCGGCCGC CTATTTCAGT GCATGTGTG), and NP (Forward: ATCTCGAGAT GGCGCTCCAA GGCACCA, Reverse: GTGCGGCCGC TTAATTGTCA TGCTCCTCC). pPRE-PB2-Flag, pPRE-PB1-Flag, pPRE-PA-Flag, and pPRE-NP-Flag were constructed by digesting pPRE-Flag plasmids with *Xho*I and *Not*I restriction endonucleases. Flag-tagged PB2, PA, PB1, and NP were constructed in expression vector pPRE with a Flag tag at the N-terminus. For construction of bimolecular fluorescence complementation (BiFC) vectors, sequences encoding the N terminal (VN, residues 1–173) and C terminal (VC, residues 174–239) fragments of Venus fluorescent protein were fused by linker (GGGSGGGS) to eIF4A3 (VN-eIF4A3) and influenza virus PB2 gene (VC-PB2), respectively. For minigenome reporter assays, vNP-luc reporter plasmid-encoded firefly luciferase, *Renilla* luciferase expression plasmid, and plasmids of pHW2000 expressing PA, PB1, PB2, and NP have been described in our previous study (Xiao et al., 2016).

### Production of Recombinant Viruses by Reverse Genetics

**FIGURE 1:**
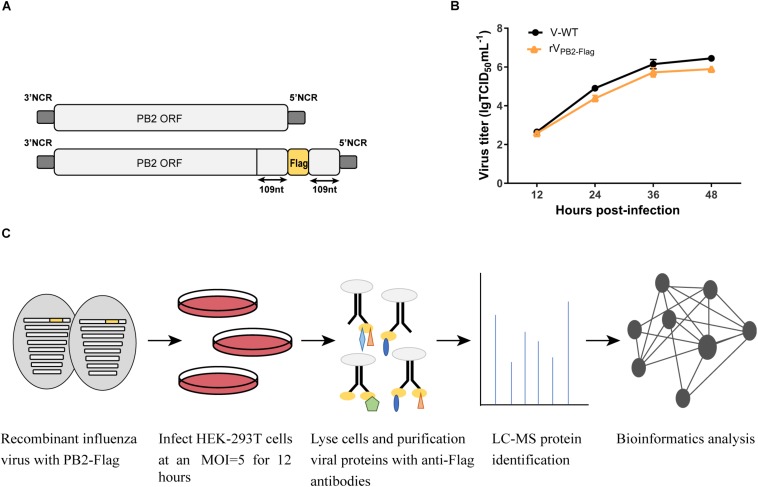
Schematic overview of the strategy to identify host factors associated with PB2 during virus infection. **(A)** Generation of the recombinant influenza virus showing the PB2 segment C-terminal fused with Flag tag. **(B)** Growth kinetics of WT virus (V) and PB2-Flag virus (rV_PB2–Flag_) in MDCK cells infected at an MOI of 0.001. **(C)** Schematic diagram of the systematic analysis of host proteins interacting with PB2 during virus infection. HEK-293T cells were infected with recombinant influenza virus rV_PB2–Flag_ at an MOI of 5. At 12 hpi, infected cells were harvested and subjected to a pulldown assay with anti-Flag affinity gel. Purified protein samples were subjected to LC-MS protein identification followed by bioinformatics analysis.

The A/Chicken/Guangdong/V/2008 (H9N2) PB2-Flag virus (rV_PB2–Flag_) was produced by reverse genetics using the pHW2000-PB2 construct (Hoffmann et al., 2000). Briefly, three tandem Flag-Tag (DYKDDDDK)- encoding sequence (GACTACAAGG ACGACGATGA CAAG) was fused to the last amino acid of the PB2 open reading frame (ORF) into the pHW2000-PB2 by standard overlapping PCR. To ensure the integrity of packaging signals, the last 109 nt nucleotide sequence of PB2 ORF was fused to the end of Flag-Tag (Figure 1A). An eight-plasmid reverse genetics system was utilized for rescuing the viruses (Yu et al., 2017).

### IP-FLAG and MS Analysis

HEK-293T cells were infected with Flag-tagged PB2 recombinant virus (rV_PB2–Flag_) at an MOI of 5. At 12 hpi, HEK-293T cells were lysed with the lysis buffer provided in the FLAG-IP kit (Sigma) according to the manufacturer’s instructions. Cell lysates were incubated with the anti-FLAG M2 affinity gel overnight at 4°C. Affinity gel was collected by centrifugation and washed three times with TBS buffer and resuspended in SDS loading buffer. Immunoprecipitation of the elution was performed by heating at 95°C for 5 min. Purified protein samples were separated with an 8% polyacrylamide gel and subjected to Coomassie blue staining. Gel slices were subjected to in-gel digestions followed by MALDI-TOF MS analysis that was performed by Beijing Genomics Institute (Shenzhen, China).

### Bimolecular Fluorescence Complementation (BiFC) Assay

The BiFC assay was performed according to a previous study (Ma et al., 2018). Briefly, HEK-293T cells grown in 24 well plates were transiently co-transfected with combinations of plasmids VC-PB2 (500 ng) and VN-eIF4A3 (500 ng), or VC-PB2 (500 ng) and VN-vector (500 g) and incubated at 37°C for 24 h. Fluorescence emission was detected using a fluorescence microscope.

### Co-immunoprecipitation

HEK-293T cells grown in 100 mm culture dishes were transiently co-transfected with combinations of indicated amounts of pPRE plasmids coding for PB2-Flag and eIF4A3-Strep, PB2-Flag and EV-Strep, or EV-Flag and eIF4A3-Strep. Twenty-four hours after transfection, cells were lysed in 500 μl ice-cold lysis buffer (Sigma) and centrifuged at 12,000 *g* for 15 min. The clarified lysates were incubated with Anti-Flag M2 affinity gel or Anti-Strep affinity gel at 4°C for 8 h. Affinity gel was collected by centrifugation and washed three times with TBS buffer and resuspended in SDS loading buffer. Elution of co-immunoprecipitates was performed by heating at 95°C for 5 min, and samples were analyzed by SDS-PAGE and western blotting.

### Indirect Immunofluorescent Assay (IFA) and Confocal Microscopy

HeLa or A549 cells were grown on cover slips in 24-well plates. After the corresponding experimental treatment, cells were fixed in 4% paraformaldehyde for 30 min at room temperature. Subsequently, permeabilized and blocked with PBS containing 0.5% Triton-X-100 and 5% bovine serum albumin for 1 h at room temperature. For immunostaining, samples were incubated with antibody against the indicated proteins for 2 h at room temperature or 4°C overnight, followed by incubation with anti-mouse IgG FITC-conjugated antibody or anti-rabbit IgG Alexa Fluor 594-conjugated antibody for 1 h, and cells nuclei were visualized with 4′,6-diamidino-2-phenylindole (DAPI, Invitrogen). All fluorescence images were acquired on an Olympus confocal microscope.

### siRNA Transfection and Virus Replication

Small interfering RNA (siRNA) targeting eIF4A3 (AGTGGAATTC GAGACCAGC, CAATCAAGCA GATCATCAA, and GCTGATGAAA TGTTGAATA) or no-target control siRNA (siNC) were purchased from Ribo Bio (Guangzhou, China). HEK-293T cells or A549 cells in 12-well plates were transfected with 50 nM of siRNA using the Lipofectamine RNAiMAX transfection reagent (Invitrogen) according to the manufacturer’s instructions, and a pool of three eIF4A3 siRNAs was used. Since the siRNA pool mediated the most efficient eIF4A3 knockdown, we used this in all subsequent experiments. Cell viability was monitored by CellTiter 96 AQueous One Solution Cell Proliferation Assay kit (Promega). Down-regulation of siRNA-targeted genes was checked by RT-qPCR and western blotting.

Multicycle replication assays were performed in HEK-293T cells and A549 cells, respectively. HEK-293T cells were infected at an MOI of 0.01, and A549 cells were infected at an MOI of 0.1 for 1 h at 37°C on 12-well plates. After 1 h viral adsorption, the cells were washed twice with PBS and then incubated with DMEM supplemented with 0.2% BSA and 1ug/ml TPCK-trypsin at 37°C. Culture supernatants were collected at 12, 24, 36, and 48 hpi. The virus titers were determined by performing 50% tissue culture infective dose (TCID_50_) assays in MDCK cells (Ma et al., 2015).

### Minigenome Reporter Assays

The polymerase activity assay was performed as described in our previous works (Xiao et al., 2016). Briefly, HEK-293T cells in a 12-well plate were co-transfected with plasmids expressing PA, PB1, PB2, NP, negative-vNP-luciferase reporter and *Renilla* luciferase reporter (200, 200, 200, 400, 200, 20 ng, respectively) and incubated at 37 °C for 24 h. Luciferase production was assayed using the dual-luciferase reporter assay system (Promega). Polymerase activity was normalized by *Renilla* expression.

### Subcellular Fractionation

RNA was separated using a PARIS kit (AM1921, Thermo Fisher). Briefly, collect 10^6^ infected A549 cells, wash once in PBS, and place washed cells on ice. Resuspend cells in 500 μL ice-cold cell fractionation buffer and incubate on ice 5∼10 min. Centrifuge samples 5 min at 4°C and 500 g. Carefully aspirate the cytoplasmic fraction away from the nuclear pellet, and then lyse nuclear pellet in cell disruption buffer. Cytoplasmic and nuclear RNAs were extracted according to the manufacturer’s instructions. A portion of each fraction was analyzed by Western blot using antibodies specific for GAPDH or Histone H2B to monitor the purity of the cytoplasmic and nuclear fractions, respectively.

### RNA Purification, Reverse Transcription and qPCR

Total RNA was extracted using the Eastep super total RNA extraction kit (Promega). The isolation of Poly(A)+ RNA was performed using the PolyATtract mRNA Isolation System (Promega) using total RNA. Nuclear and cytoplasmic Reverse transcription was performed using Moloney murine leukemia virus reverse transcriptase (M-MLV, Takara) with either an oligo(dT) primer or a strand-specific primer. Strand-specific primer sequences used in this study are listed as follows (5′-3′): PB2 cRNA (GCTAGCTTCA GCTAGGCATC AGTAGAAACA AGGTCGTT), and PB2 vRNA (GGCCGTCATG GTGGCGAATA ATGCGTGACA TACTGGGAAC). Quantitative real-time PCR (qPCR) was carried out using a GoTaq qPCR master mix (Promega) on an Applied Biosystems 7300 plus qPCR cycler. The following primers were used (5′-3′): 18S rRNA (Forward: CAAGACGGAC CAGAGCGAAA, Reverse: GGCGGGTCAT GGGAATAAC) (Wisskirchen et al., 2011); NP (Forward: GCACCAAACG ATCTTATGAGC, Reverse: CTGTATGTAG AACCTTCCGAT); PB2 mRNA (Forward: AATGCGTGAC ATACTGGGAAC, Reverse: CCCCTTAGTA CCGCAGACTC C); PB2 cRNA (Forward: GCTAGCTTCA GCTAGGCATC, Reverse: GGAG TCTGCG GTACTAAGGG G); PB2 vRNA (Forward: GGCCGTCAT GGTGGCGAAT, Reverse: CCCCTTAGTA CCG CAGACTC C); NS1 (Forward: TGATGCCCCA TTTCTAGACC, Reverse: ATCTGCTCCA CTATATGCTT); NS2 (Forward: CAAGCTTCCA GGACATACTG AT, Reverse: TTCTCCAAGC GAATCTCT); M1 (Forward: AAGTTGCACT CAGTTACTCA, Reverse: TTCTGTAGTT ACCGTTCCCA); M2 (Forward: CGAGGTCGAA ACGCATACCA G, Reverse: AACCGTATTT AAAGCGACGA); GAPDH (Forward: CGCTGAGTAC GTCGTGGAGT C, Reverse: GCAGGAGGCA TTGCTGATGA) (Hawkins et al., 2009).

### Statistics

Multiple comparisons were performed by using an unpaired *t*-test and analysis of variance (ANOVA) in the GraphPad Prism software (GraphPad Software Inc.). The ^*^P-value < 0.05, ^∗∗^*P*-value < 0.01 was considered significant.

## Results

### Identification of eIF4A3 as an Interactor of Avian Influenza Virus PB2 in a Pulldown Screen

Our previous studies have identified a mammalian-adapted field avian influenza virus, A/Chicken/Guangdong/V/2008 (H9N2, V), that is capable of efficient replication in mammalian cells and is highly pathogenic in mice (Li et al., 2012). To identify host factors associated with PB2 that are essential for efficient AIV replication in human cells, we constructed a recombinant influenza A/Chicken/Guangdong/V/2008 (H9N2) PB2-Flag virus (rV_PB2–Flag_) with the PB2 C-terminal fusing three tandem Flag epitopes by reverse genetics technology (Figure 1A). The rV_PB2–Flag_ virus displayed similar growth kinetics to that of a wild-type virus on MDCK cells (Figure 1B), suggesting the functional activity of the recombinant proteins. To globally identify host cellular factors that interact with the PB2 protein, we infected the HEK-293T cells with the rV_PB2–Flag_ virus and purified reconstituted viral PB2 using the Flag antibodies coupled with affinity gel and identified the co-purified factors by liquid chromatography-mass spectrometry (LC-MS) (Figure 1C). Uninfected HEK-293T cells were used as a control. The LC-MS analysis identified viral PB2, PB1, PA, and NP proteins. Additionally, 80 cellular proteins, which fall into different functional categories, were also identified (Supplementary Table S1). eIF4A3 was present specifically in complex with Flag-PB2-proteins in three times replications.

### Interaction of eIF4A3 With AIV PB2 and Other Subunits of vRNP

**FIGURE 2:**
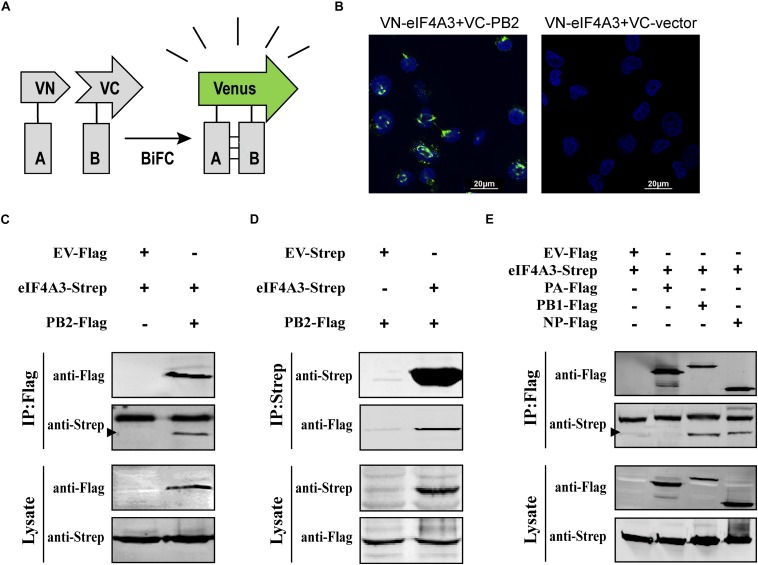
eIF4A3 interacts with AIV PB2 and other subunits of viral polymerase in mammalian cells. **(A)** Schematic representation of BiFC analysis. Gray rectangle A and B represent a pair of proteins detected by BiFC. VN and VC represent the N-terminal fragment (residues 1–173) and C-terminal fragment (residues 174–239) of Venus protein, respectively. **(B)** BiFC assay for detecting interaction between PB2 and eIF4A3. HEK-293T cells were transiently transfected with indicated plasmids and incubated for 24 h. Fluorescence emission and brightfield were visualized. **(C,D)** Co-immunoprecipitation assays were performed with FLAG-tagged PB2 and Strep-tagged eIF4A3. Strep-tagged eIF4A3 or Strep-tagged empty vector was co-transfected in HEK-293T cells with Flag-tagged PB2 or Flag-tagged empty vector. After 24 h, cells were lysed followed by immunoprecipitation (IP) and western blot analyses. The arrow indicates the position of immunoblot. **(E)** The control vector, Flag-tagged PA, PB1, or NP expression constructs were individually transfected into HEK-293T cells along with Strep-eIF4A3 plasmid. After 24 h, cells were lysed followed by immunoprecipitation (IP) and western blot analyses.

To confirm that eIF4A3 directly interacted with PB2, a Venus-based BiFC system was performed (Figure 2A; Kerppola, 2006; Zhu et al., 2013). VN-eIF4A3 was co-transfected into HEK-293T cells with VC-PB2 or VC-vector. At 24 h post-transfection, obvious fluorescence signals were observed in VN-eIF4A3 and VC-PB2 co-transfected cells, whereas no fluorescent emission was observed in cells transfected with a combination of VN-eIF4A3 and VC-Vector (Figure 2B). These results suggested a direct interaction between eIF4A3 and PB2. To further validate the specificity interaction of eIF4A3 and PB2, we performed a co-immunoprecipitation (CO-IP) assay. Strep-tagged eIF4A3 or Strep-tagged empty vector was co-transfected in HEK-293T cells with Flag-tagged PB2 or Flag-tagged empty vector. The interaction of eIF4A3 and PB2 was examined by precipitating eIF4A3 or PB2 from the lysates. At 24 h post-transfection, the cell lysates were subjected to IP using anti-Flag or anti-Strep antibody coupled affinity gel, followed by western blotting using anti-Flag and anti-Strep antibodies. eIF4A3 was detected in the precipitated complex when Flag-PB2 was expressed, but not when Flag-vector was expressed (Figure 2C). Similarly, PB2 was detected in the precipitated complex, when Strep-eIF4A3 was transfected (Figure 2D). The data demonstrate that there was a direct interaction between eIF4A3 and PB2. The RNP complex of AIV was constituted by RNA, NP, and the RdRP (PB2, PB1, and PA); thus, we want to address the specificity of the interaction between eIF4A3 and other RNP subunits. Strep-tagged eIF4A3 was co-transfected in HEK-293T cells with Flag-tagged PA, PB1, NP, or an empty vector. At 24 h post-transfection, the cell lysates were subjected to IP with Flag antibodies, followed by western blotting using anti-Flag and anti-Strep antibodies. We found that eIF4A3 also co-purified with PB1 and NP, but there was no interaction between eIF4A3 with PA (Figure 2E). Together, our results indicated that eIF4A3 also interact with AIV PB1 and NP; therefore, there is a direct interaction between eIF4A3 and AIV RNP complex.

**FIGURE 3:**
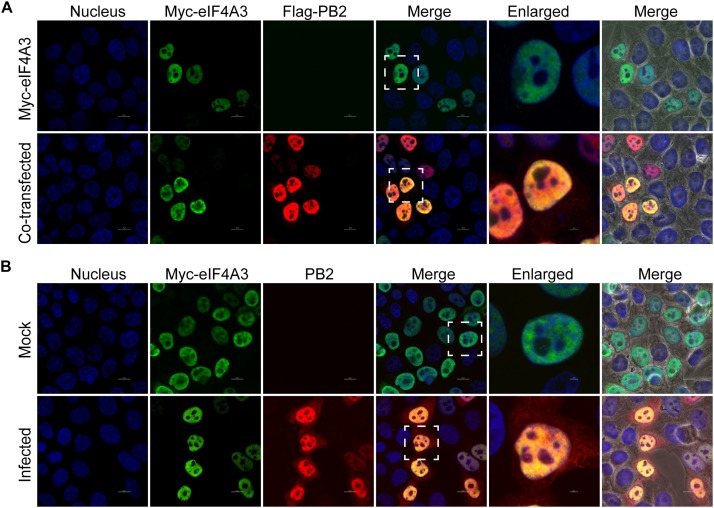
Subnuclear localization of eIF4A3 and PB2. **(A)** Colocalization of eIF4A3 and AIV PB2 in transfected cells. HeLa cells were co-transfected with Myc-tagged eIF4A3 and Flag-tagged PB2 or Flag-tagged empty vector. After 24 h post-transfection, co-transfected cells were fixed and stained for Myc-tagged eIF4A3 (green) and Flag-tagged PB2 (red) using the anti-Myc mouse antibodies and anti-Flag rabbit antibodies, followed by immunostaining with the secondary antibodies. **(B)** Colocalization of eIF4A3 and AIV PB2 in infected cells. HeLa cells were transfected with Myc-tagged eIF4A3. After 24 h post-transfection, cells were infected with V virus at an MOI of 1 or mock infected and fixed after 12 h post-infection, then stained for Myc-tagged eIF4A3 (green) and PB2 (red) using the anti-Myc mouse antibodies and anti-PB2 rabbit antibodies, followed by immunostaining with the secondary antibodies. Nuclei were stained with DNA-binding dye DAPI (blue).

Our BiFC and IP experiments clearly demonstrate the interaction between viral PB2 and eIF4A3. Therefore, we were interested in studying where the interaction between PB2 and eIF4A3 could occur in transfected or virus-infected cells. HeLa cells were co-transfected with Myc-tagged eIF4A3 and Flag-tagged PB2 or Flag-tagged empty vector. At 24 h post-transfection, co-transfected cells were fixed and stained for Myc-tagged eIF4A3 (green) and Flag-tagged PB2 (red) using the anti-Myc mouse antibodies and anti-Flag rabbit antibodies, followed by immunostaining with the secondary antibodies. Confocal microscopy showed that Myc-tagged eIF4A3 was localized mainly in the nucleus, Flag-PB2 also expressed mainly in the nucleus, and obvious co-localization between Myc-eIF4A3 and Flag-PB2 was observed in the nucleus (Figure 3A). We next evaluated the subcellular localization of eIF4A3 and PB2 in infected cells. HeLa cells were transfected with Myc-tagged eIF4A3. At 24 h post-transfection, cells were infected with V virus at an MOI of 1 or mock infected and fixed after 12 h post-infection (hpi), then stained Myc-tagged eIF4A3 (green) and PB2 (red) using the anti-Myc mouse antibodies and anti-PB2 rabbit antibodies, followed by immunostaining with the secondary antibodies. In infected cells, eIF4A3 was also mainly localized in the nucleus. Interestingly, the proportion of PB2 in the cytoplasm increased compared to transfected cells, but PB2 is still mainly concentrated in the nucleus and co-localized with Myc-eIF4A3 (Figure 3B). Taken together, these data suggest that eIF4A3 partially co-localized with PB2 in the nucleus.

### Endogenous eIF4A3 Is Required for Efficient Influenza Virus Replication

**FIGURE 4:**
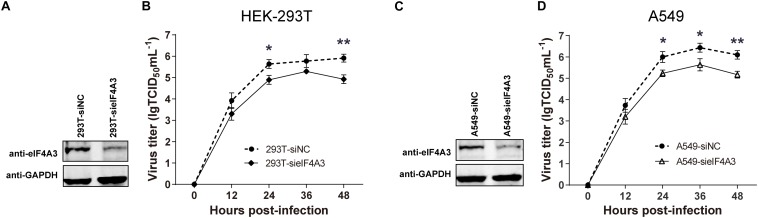
Knockdown endogenous eIF4A3 impairs influenza virus replication. HEK-293T cells were transfected with negative control (NC) siRNA or sieIF4A3. **(A)** Cells were harvested at 36 h post-transfection to evaluate eIF4A3 protein knockdown efficiency by immunoblot analysis. **(B)** Thirty-six hours after post-transfection of siRNA, NC and eIF4A3 knockdown cells were infected with influenza A/Guangdong/V at an MOI of 0.01, and then the resulting infectious viral particles were harvested and subjected to TCID_50_ analyses on MDCK cells at 12, 24, 36, and 48 hpi. A549 cells were transfected with eIF4A3 or NC siRNA. **(C)** Cells were harvested at 36 h post-transfection to evaluate eIF4A3 protein knockdown efficiency by immunoblot analysis. **(D)** Thirty-six hours after post-transfection of siRNA, negative control and eIF4A3 knockdown cells were infected with influenza A/Guangdong/V at an MOI of 0.1, and then the resulting infectious viral particles were harvested and subjected to TCID_50_ analyses on MDCK cells at 12, 24, 36, and 48 hpi. The data are shown as means ± SD (*n* = 3) and the significance was calculated using an unpaired *t*-test (^*^p < 0.05; ^∗∗^*p* < 0.01).

To examine the functional contribution of eIF4A3 to the viral life cycle, we performed siRNA-mediated eIF4A3 knockdown in HEK-293T and A549 cells. HEK-293T and A549 cells were treated with eIF4A3 or negative control (NC) siRNA. At 36 h post-transfection, cells were infected with influenza virus V at an MOI of 0.01 and 0.1, respectively, and then the production of infectious viral particles was subjected to TCID_50_ analyses on MDCK cells at 12, 24, 36, and 48 hpi. The amount of eIF4A3 was reduced more-than 70% in cells transfected with eIF4A3 siRNA compared to that treated with NC siRNA (Figures 4A,C). At 24 and 48 hpi, the virus titers were remarkably reduced in eIF4A3-knockdown HEK-293T cells by about 5.5 to 9.7-fold over controls (Figure 4B). Similar reductions were observed on A549 cells at 24, 36, and 48 hpi (about 5.8 to 8.5-fold) (Figure 4D). Together, these results indicated that endogenous eIF4A3 is essential for AIV multiplication in cell culture.

### eIF4A3 Regulates Influenza Viral RNA Synthesis Machinery

**FIGURE 5:**
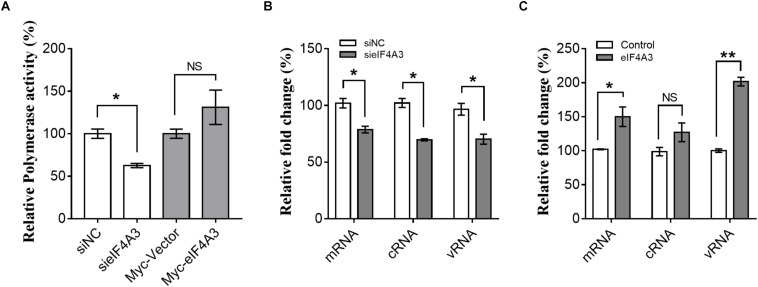
Role of eIF4A3 during the replication cycle of influenza virus. **(A)** HEK-293T cells treated with eIF4A3 siRNA or overexpress plasmids. At 36 h post-transfection, cells were transfected with firefly minigenome reporter and *Renilla* expression control. **(B)** Cultures of HEK-293T cells were transfected with eIF4A3 or NC siRNA. Thirty six hours after post-transfection, cells were infected with influenza A/Guangdong/V at an MOI of 1. Relative vRNA, cRNA, and mRNA expression of segment 1 was measured by strand-specific real-time RT-qPCR after 24 hpi. The expression of vRNA, cRNA, and mRNA in siNC cells was set to 1. **(C)** eIF4A3 or empty vector were expressed for 24 h before being infected with influenza A/Guangdong/V at an MOI of 1 and then incubated at 37°C for 24 h before reverse transcription followed by qRT-PCR for vRNA, cRNA, and mRNA of segment 1. The data are shown as means ± SD (*n* = 3) and the statistical significance of differences from the data obtained for control cells was determined using the Student *t-*test (^*^p < 0.05; ^∗∗^*p* < 0.01).

The observations described above suggest that the interaction of viral RNP with eIF4A3 might serve to recruit cellular RNA helicase into viral polymerase to assist directly or indirectly in viral RNA replication. To study this possibility, we examined the role of eIF4A3 in viral polymerase activity and RNA synthesis in infected cells by minireplicon assay and specific viral mRNA, cRNA, and vRNA RT-qPCR assay. HEK-293T cells treated with eIF4A3 siRNA or overexpress plasmids for 36 h were transfected with expression plasmids encoding influenza NP, PA, PB1, and PB2 proteins. The vRNA-like minigenome is encapsidated by NP and recognized, transcribed, and replicated by the polymerase proteins. Firefly luciferase activities were obtained after 24 h post-transfection. Our results showed that the depletion of eIF4A3 significantly inhibited viral polymerase activity in human cells (Figure 5A). There are three different types of RNA in the process of influenza virus replication, namely, mRNA, cRNA, and vRNA. The mRNA and cRNA are very similar, so we designed special reverse transcription and detection primers according to previous studies to distinguish them (Kawakami et al., 2011; Long et al., 2016). qPCR analysis revealed that the levels of viral PB2 segment mRNA, cRNA and vRNA were decreased in eIF4A3-knockdown cells (Figure 5B). To confirm the role of eIF4A3 in viral RNA synthesis, eIF4A3 was also overexpressed in virus-infected cells. This led to significant enhancement of viral mRNA and vRNA levels (Figure 5C). Taken together, these data indicate that viral RNP recruits cellular endogenous RNA helicase eIF4A3 to promote viral polymerase activity and then affects RNA synthesis.

### Knockdown of eIF4A3 Reduces the NS2/NS1 M2/M1 mRNA and Protein Ratios in Infected Cells

**FIGURE 6:**
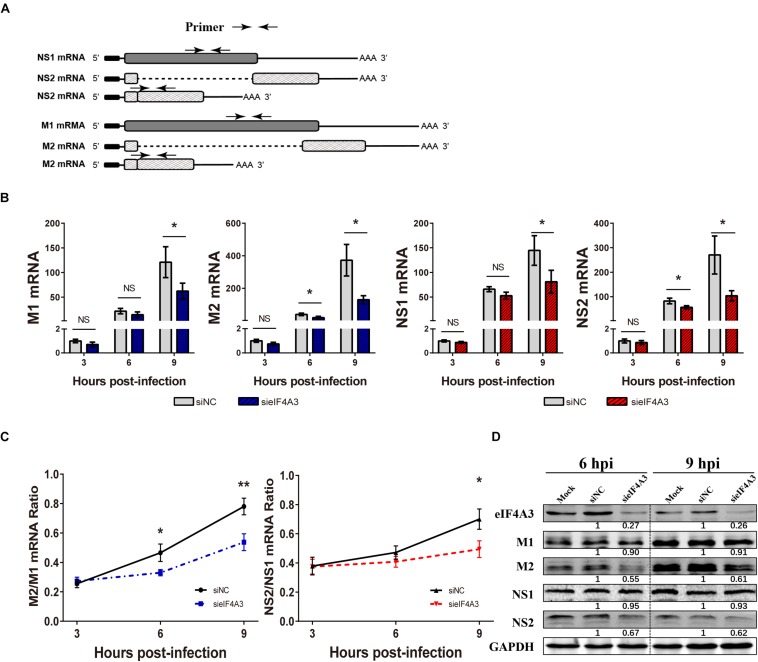
Effect of eIF4A3 knockdown on the accumulation of influenza virus mRNAs and proteins. **(A)** Schematic representation of the primers used for RT-qPCR. M1 mRNA along with its alternatively spliced products M2 mRNA, NS1 mRNA and its alternatively spliced product NS2 mRNA, is depicted. The arrowheads show primer positions for detection of various mRNAs in the following experiments. **(B)** A549 cells were treated with negative control (NC) or eIF4A3 siRNA for 36 h and infected with influenza virus V at an MOI 2 for the indicated time points. The NS1, NS2, M1, and M2 mRNA copy numbers were determined at the indicated times post-infection by strand specific RT-qPCR and were normalized to the level of the same mRNA species at 3 hpi in cells treated with the NC siRNA. The data are expressed as the mean ± SD of three independent experiments and the significance was calculated using an unpaired *t*-test (^*^p < 0.05). **(C)** The ratios of NS2/NS1 and M2/M1 mRNA levels in eIF4A3-silenced or negative control cells. The data are shown as means ± SD (*n* = 3) and the significance was calculated using an unpaired *t*-test (^*^p < 0.05; ^∗∗^*p* < 0.01). **(D)** A549 cells treated with eIF4A3 or NC siRNA were infected with influenza virus V at an MOI of 5. At 6 and 9 hpi, cell lysates were subjected to western blot analysis using antibodies for the eIF4A3, M1, M2, NS1, NS2, and GAPDH proteins. GAPDH was used as loading control.

For influenza viruses, two of the eight segments produce both unspliced and spliced mRNA transcripts. The M segment gives rise to unspliced M1 mRNA and spliced M2 mRNA; NS segment produces the unspliced NS1 mRNA and the spliced NS2/NEP transcript (Dubois et al., 2014). As a novel EJC core component, eIF4A3 plays an important role in spliced pre-mRNA near exon-exon junctions (Sauliere et al., 2012). We hypothesized that the viral replication defect in eIF4A3-depleted cells could also be due to impaired splicing of viral mRNA. We thus examined the effect of eIF4A3 depletion on the accumulation of viral mRNA. To determine if eIF4A3 facilitates the viral mRNA’s splicing process, we first examined the effect of eIF4A3 depletion on the accumulation of viral M1, M2, NS1, and NS2 mRNA levels. A549 cells were transfected with eIF4A3 or NC siRNA and then infected with influenza virus V at an MOI of 2. At 3, 6, 9 hpi, total cell RNA was extracted and Poly(A)+ mRNA was purified from it. Virus mRNA levels were measured by RT-qPCR using specific primers (Figure 6A). Indeed, the accumulation of the M1, M2, NS1, and NS2 mRNA decreased in cells depleted of eIF4A3, particularly so at the latest time-point (Figure 6B). The ratio of NS2 over NS1 mRNA showed a stable decrease at 9 hpi, and the ratio of M2 over M1 mRNA was reduced at 6 and 9 hpi, indicating a splicing defect (Figure 6C). The effect of eIF4A3 knockdown on the accumulation of viral M1, M2, NS1, and NS2 proteins was then examined. A549 cells treated with eIF4A3 or NC siRNA were infected with influenza virus V at an MOI of 5. The accumulation of viral proteins was monitored by western-blot analysis of cell lysates, using antibodies for the eIF4A3, M1, M2, NS1, NS2, and GAPDH proteins. The ratio of M2 over M1 protein remained stable except for a two-fold reduction in eIF4A3-silenced cells at 6 and 9 hpi, and the NS2 over NS1 protein ratio also decreased significantly at 6 and 9 hpi (Figure 6D). Overall, these data indicated that the production of spliced M2 and NS2 mRNA was specifically impaired when eIF4A3 was depleted.

### eIF4A3 Contributes to the Nuclear Export of Viral Transcripts

**FIGURE 7:**
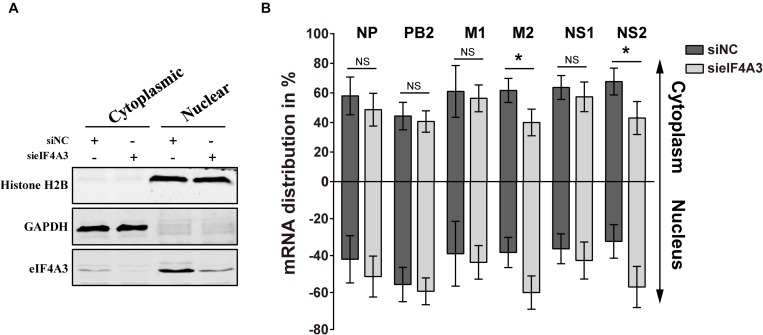
Reduced export of viral spliced mRNA in cells treated with eIF4A3 specific siRNA upon infection with AIV. A549 cells were treated with the NC (dark gray bars) or eIF4A3 (light gray bars) siRNAs for 36 h and subsequently infected with influenza virus V at an MOI of 5 for 4 h. Cytoplasmic and nuclear extracts were isolated and poly(A)+ mRNAs were purified. **(A)** The purity of the cytoplasmic and nuclear fractions was monitored by Western blot using antibodies specific for GAPDH or Histone H2B, respectively. **(B)** The levels of viral mRNA were quantified by RT-qPCR, normalized to 18srRNA. The data are shown as means ± SD (*n* = 3) and the significance was calculated using an unpaired *t*-test (^*^p < 0.05).

Previous research has reported that after pre-mRNA splicing is completed, EJCs remain deposited on mRNA when transported to the cytoplasm, which could facilitate mRNA export (Singh et al., 2012). Thus, we want to verify whether eIF4A3 plays a role in regulating the nuclear export of viral spliced mRNA (NS2 and M2) in the virus-infected cells. To this end, siRNA-treated A549 cells were infected with influenza virus V at an MOI of 5. After 4 hpi, infected cells were subjected to subcellular fractionation. The purity of the cytoplasmic and nuclear fractions was monitored by Western blot using antibodies specific for GAPDH or Histone H2B, respectively (Figure 7A). The levels of cytoplasmic and nuclear PB2, NP, M1, M2, NS1, and NS2 mRNA were then determined by RT-qPCR (Figure 7B). In eIF4A3-silenced cells, the proportions of cytoplasmic viral M2 and NS2 mRNAs were reduced (10 to 15%), whereas the intronless viral mRNA (PB2, NP, M1, and NS1) nucleo-cytoplasmic mRNA ratio showed no change. These data indicated that eIF4A3 also plays an essential role in mediating AIV-spliced mRNA efficient nuclear export.

### Knockdown of eIF4A3 Impairs the Nuclear Export of vRNP in Infected Cells

**FIGURE 8:**
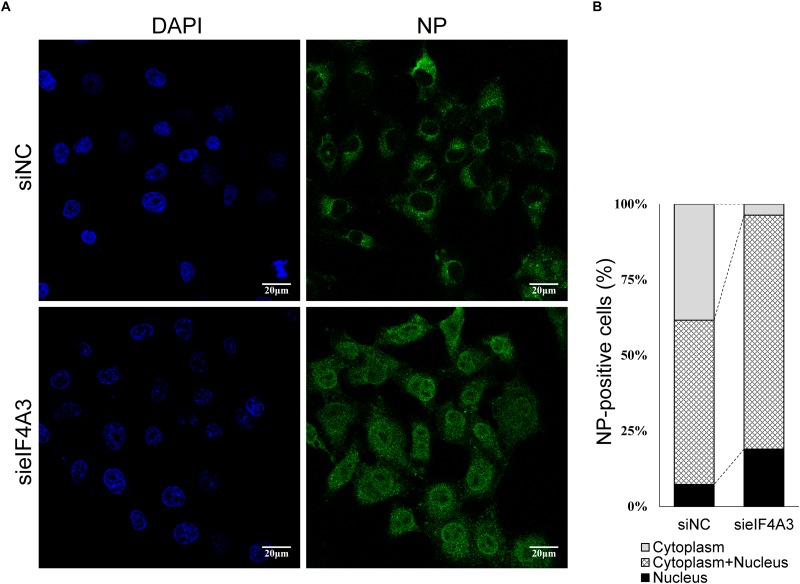
eIF4A4 knockdown accumulates viral NP proteins in the nucleus. **(A)** A549 cells were transfected with NC or eIF4A3 siRNA for 36 h and infected with influenza virus V at MOI = 5. After 5 hpi, cells were fixed and analyzed by immunofluorescent staining with anti-NP antibody, followed by Alexa Fluor 488 goat anti-mouse antibody. Nuclei were counterstained with DAPI, and the cells were visualized under an Olympus laser scanning confocal fluorescence microscope. **(B)** Percentage of cells with different NP localization.

The Influenza NS2/NEP protein is known to act as an adapter during Crm1 (chromosome region maintenance 1)-mediated influenza virus vRNPs nucleus export, thereby ensuring that the viral vRNP is available for packaging into progeny virions on the cellular periphery (Paterson and Fodor, 2012). We thus examined whether, in cell depleted eIF4A3, the synthesis of NS2 reduction was associated with a defect of vRNP nuclear export. As we know, vRNP is composed of a large amount of NP proteins entangled by viral RNA, so monitoring the condition of NP can reflect the change of vRNP (Ando et al., 2016). A549 cells were transfected with eIF4A3 or NC siRNA and then infected with influenza virus V at an MOI of 5. Intracellular localization of viral NP protein was monitored at 5 hpi by indirect immunofluorescence. In NC siRNA-treated cells, NP protein localized mainly in the cytoplasm, whereas in cells knockdown of the eIF4A3 gene, there was an obvious accumulation of NP in the nucleus (Figure 8A). To confirm the change in location, the quantification of the NP localization on average of 140 cells in each experimental condition demonstrated that eIF4A3 depletion inhibited vRNP transferred from the nucleus to the cytoplasm (Figure 8B). Altogether, these results suggest that the depletion of eIF4A3 impairs efficient AIV vRNP nuclear export.

## Discussion

Host cellular machinery plays an indispensable role in the influenza A virus life cycle. Numerous systems-level datasets designed to dissect the interaction between host factors and influenza virus proteins have been reported (York et al., 2014; Heaton et al., 2016; Yang et al., 2018), aiming to provide unprecedented molecular insights into host factors in infections and to find a better therapeutic target to fight influenza infection. In this study, we identified the host cellular protein eIF4A3 RNA helicase as an interacting partner of the PB2 protein by pulldown with the assistance of PB2 Flag-tagged recombinant AIV. We confirmed the interaction of eIF4A3 with PB2, PB1, and NP proteins. Furthermore, eIF4A3 is essential for AIV RNA polymerase activity and RNA synthesis. As the EJC core, eIF4A3 shows significant function in nascent mRNA splicing and mRNA nuclear export. Thus, these findings revealed eIF4A3 as an essential factor in AIV infection that may become a novel and promising target for the development of new host-directed anti-influenza therapeutics.

During transcription, viral RNA polymerase attached directly or indirectly to host Pol II, participated in a “cap-snatching” mechanism from cellular pre-mRNA, then synthesized capped mRNA using capped RNA primer. After completion, the 5′ cap of mRNA is released from RNA polymerase and is bound by the 20 kDa subunit, the cellular nuclear cap-binding complex (CBC) triggering the recruitment of cellular factors for mRNP assembly (Fodor, 2013). Functionating of RNA polymerase is believed to depend on the participation of numerous cellular proteins, although the molecular details remain poorly characterized (Bauer et al., 2018). We showed that eIF4A3 co-purifies with PB2-Flag in infected cell lysates. Our BiFC and IP experiments clearly demonstrate the direct interaction between viral PB2 and eIF4A3, and we further confirmed that this interaction mainly occurred in the nucleus using confocal microscopy. But unexpectedly, fluorescence signals of BiFC appeared in the cytoplasm of a few cells, possibly because PB2 and eIF4A3 also play a certain role in the cytoplasm (Singh et al., 2012; Long and Fodor, 2016). In addition, we found that the fluorescence signal of BiFC was very uneven, forming many bright spots, and sometimes it was difficult to determine its specific location. In other studies, we also found a similar phenomenon (Li et al., 2016). Furthermore, we confirmed that eIF4A3 also binds to NP and viral polymerase subunits PB1. Thus, we presume that eIF4A3 could bind to the viral RNP and may be associated with the vRNA, mRNA, or cRNA process. A minireplicon assay indicated that eIF4A3 depletion resulted in a significant defect in the activities of viral RNA polymerases, which suggests that eIF4A3 directly or indirectly participated in the process of viral mRNA transcription. Regarding the process of cellular mRNA synthesis, many Pol II recruitment factors including eIF4A3, which regulate RNA capping, splicing, and polyadenylation, as well as an assembly into mRNP and its nuclear export (Bauer et al., 2018). Fodor thinks that the cellular Pol II transcriptional machinery not only facilitates the access of the viral RNA polymerase to the 5′ cap structure, but might also assist in recruitment cellular factor participate viral mRNA maturity and nuclear export (Fodor, 2013). Thus, we speculate that the cellular Pol II complex recruitment eIF4A3 interacted with viral RNA polymerase which participating in the viral mRNA process.

As a core component of the EJC, one of the main functions of eIF4A3 is to participate in the splicing of eukaryotic per-mRNA. Some research indicates that eIF4A3 also plays a role in the splicing of viral mRNA (Ziehr et al., 2016). Most of influenza viral mRNA is intronless, while segments 7(M) and 8(NS) can undergo mutable splicing to generate spliced as well as unspliced mRNA (Tsai et al., 2013). We found that the nascent mRNA of NS and M splicing is impaired in cells depleted for eIF4A3. Apparently, eIF4A3 depletion impeded the exon-exon joint function of EJC, so that it impaired the accumulation of spliced viral mRNA. A previous study suggested that the viral polymerase complex can block the M mRNA_3_ 5′ splice site to regulate the production of M2 mRNA in influenza virus M1 mRNA (Shih et al., 1995). Taken together with our protein interaction results, we cannot exclude the possibility that the depletion of eIF4A3 influenced viral polymerase complex recognition and regulation of the M1 mRNA 5′ splice site, thereby affecting the amount of M2 mRNA.

One of the primary functions of eIF4A3 is facilitating the export of spliced host mRNA from the nucleus to the cytoplasm (Moore and Proudfoot, 2009). Our study reveals that eIF4A3 also plays an essential role in mediating efficient AIV-spliced mRNA nuclear export, as eIF4A3 depletion reduces the cytoplasmic distribution of spliced viral mRNA, M2, and NS2 in the cytoplasm. The main 10 influenza A virus mRNA can be classified in three structural classes: intronless (PB2, PB1, PA, NP, HA, and NA), intron-containing but unspliced (M1 and NS1), and spliced (M2 and NS2). Different mRNA structures may have different assembly and nuclear export pathways (Read and Digard, 2010). Several previous studies have shown that there are at least two distinct cases of influenza A virus transcript export: viral intronless transcripts and spliced mRNA transcript approach (Dubois et al., 2014). Spliced viral mRNAs have a similar export mode to cellular mRNA. Since eIF4A3 binds to influenza A virus polymerase and regulates M2 and NS2 pre-mRNA splicing, a model can be proposed in which viral polymerase recruits EJC to control the alternative splicing of M1 and NS1 mRNA. After splicing, the EJC interaction with Aly/REF and NFX1 promote spliced mRNA export to the cytoplasm. Alternatively, influenza A virus could utilize eIF4A3 in an EJC-independent mRNA export mode.

In influenza virus infected cells, transcription and replication are carried out simultaneously generating large numbers of vRNPs. The NS2 protein is implicated in mediating the export of vRNPs from the host cell nucleus by directly interacting with it, thereby ensuring that the viral genomic segments are available for packaging into daughter virions on the cellular periphery (Huang et al., 2013). We have confirmed that in AIV infected cells, knockdown of the eIF4A3 results in the decrease of NS2 mRNA in the stage of synthesis and nuclear export. In agreement with these results, we found eIF4A3 depletion led to a greater defect in vRNP nuclear export. A previous study also showed a similar phenomenon, that knockdown of splicing-related protein RED or SUM1 can significantly inhibit the generation of NS2 protein, thus the nuclear export of neo-synthetized vRNPs was strongly impaired (Fournier et al., 2014). Furthermore, recent studies suggested that NS2 may also contribute to the viral budding process and the regulation of the accumulation of viral RNA species (Paterson and Fodor, 2012). Knockdown eIF4A3 can obviously disturb the viral spliced mRNA process and spur a cascade of distinct events, thereby affecting the functions of virus replication.

Moreover, eIF4A3 participates in RNA metabolism, including NMD. Cancer cells are considered highly dependent on the NMD system in avoiding the accumulation of aberrant proteins (Gilboa, 2013). Therefore, some eIF4A3 inhibitors were developed as anti-cancer agents (Mizojiri et al., 2017). Meanwhile, eIF4A family members have been proved to be essential in HCMV virus growth. A natural eIF4A family inhibitor, pateamine A, was found to inhibit HCMV replication during all stages of the virus lytic cycle (Ziehr et al., 2016). Hence, these eIF4A3 inhibitors have potential in the development of new anti-influenza drugs.

In summary, we describe that by employing a novel PB2-tagging approach, we identified influenza PB2-interacting partners during virus infection, and we demonstrated that host factor eIF4A3 works as a binding protein of the AIV ribonucleoprotein complexes in mammalian cells. This interaction was shown to be important for the effective multiplication of the influenza virus, as siRNA knockdown of eIF4A3 expression resulted in the downregulation of viral polymerase activity and RNA synthesis. We further demonstrate that eIF4A3 is required for viral intron mRNA splicing and efficient spliced mRNA nuclear export. Our study revealed the viral polymerase recruits eIF4A3 to participate in spliced mRNA maturation; therefore, the host RNA helicase eIF4A3 could serve as a novel target for drug development against influenza virus infection.

## Data Availability

All datasets generated for this study are included in the manuscript and/or the Supplementary Files.

## Author Contributions

WQ and ML conceived and designed the experiments. XR, YY, HL, and JH performed the experiments. XR, YY, HL, and WQ analyzed the data. AZ, SL, PH, and BL contributed to reagents, materials, and analysis tools. XR, YY, and WQ wrote the manuscript. All authors read and approved the final manuscript.

## Conflict of Interest Statement

The authors declare that the research was conducted in the absence of any commercial or financial relationships that could be construed as a potential conflict of interest.
